# The incidence of oral cavity cancer in Iran: A systematic review and meta‐analysis

**DOI:** 10.1002/cnr2.1836

**Published:** 2023-05-16

**Authors:** Mohammad Jokar, Negin Namavari, Seyed Alireza Moshiri, Hossein Kargar Jahromi, Vahid Rahmanian

**Affiliations:** ^1^ Faculty of Veterinary Medicine, Karaj Branch Islamic Azad University Karaj Iran; ^2^ School of Medicine Jahrom University of Medical Science Jahrom Iran; ^3^ Research Center for Non‐Communicable Disease Jahrom University of Medical Sciences Jahrom Iran; ^4^ Department of Public Health Torbat Jam Faculty of Medical Sciences Torbat Jam Iran

**Keywords:** epidemiology, incidence, Iran, oral cancer

## Abstract

**Background and Aims:**

Oral cancer is now a top priority for non‐communicable illnesses and universal health care plans, according to the WHO. There is no general estimate of the incidence of oral cavity cancer in Iran, despite multiple investigations. The purpose of this study is to evaluate the age‐standardized incidence rate (ASR) of oral cavity cancers in Iran.

**Method:**

In accordance with the MOOSE (Meta‐analyses of Observational Studies in Epidemiology) Checklist recommendations, this systematic review was conducted. PubMed/MEDLINE, Web of Science, ScienceDirect, Embase, Scopus, ProQuest, and Google Scholar were used as the international databases for the systematic literature search, while SID (Scientific Information Database), Magiran and element were used as the Iranian databases. The heterogeneity of the research will be evaluated by means of the inverse variance and Cochran *Q* tests, along with random‐effect models. It was determined what caused the heterogeneity using a meta‐regression model. By eliminating experiments one at a time, sensitivity analysis was used. The meta‐analysis was corrected utilizing the Trim‐and‐fill method due to the identification of noteworthy publication bias via the Egger's test and asymmetry of the funnel plot.

**Results:**

This research incorporated a total of 22 journal articles. The pooled ASR of oral cavity cancer for males and females was estimated at 1.96 (95% CI: 1.65–2.26) (*Q* statistic = 1118.09, df = 25, *p* < .0001, *I* ^2^ = 97.8%), and 1.46 (95% CI: 1.14–1.77) (*Q* statistic = 2576.99, df = 26, *p* < .0001, *I* ^2^ = 99.0%), respectively. According to the funnel plots and Egger's test, there is no evidence of publication bias in studies reporting on males (bias = 1.3220, 95% CI: −3.9571, 6.6012, *p* = .610), but for ASR in females, Egger's test was significant (bias = −7.6366, 95% CI: 2.2141, 13.05904, *p* = .008). Based on Trim‐and‐fill methods, overall ASR corrected in females was estimated to be 1.36 (95% CI: 1.05%–1.66%).

**Conclusion:**

Iran's oral cavity cancer incidence was lower than the global average, but owing to variables including an aging population, a rise in life expectancy, and exposure to risk factors like smoking, we anticipate an increasing trend.

## 1 INTRODUCTION

Cancer is one of the leading causes of mortality globally. Globally, it was predicted that 10.3 million people will die from cancer in 2020 and 19.3 million people would get the disease. Oral malignancies account for around 3.2% of all cancer‐related fatalities.^1^ Oral cancer is now a top priority for non‐communicable diseases and universal health coverage programs, according to the World Health Organization (WHO).^2^ Oral cancer incidence and fatality rates vary considerably around the globe. Developing nations, particularly those in South Asia, including India, Pakistan, and Bangladesh, have the highest incidence of oral cancer.^3^, ^4^

The primary risk factors for oral cavity cancer include age, male gender, tobacco and alcohol consumption, exposure to UV radiation, immunosuppression (such as from HIV infection or organ transplantation), and human papillomavirus (HPV) infection.^5^, ^6^, ^7^, ^8^ In Iran, cancer is currently the second leading cause of death, resulting in over 70 000 deaths each year.^9^ According to GLOBOCAN 2020, there are 6 oral and 2.3 lip cavities per 100 000 individuals in the globe for men and women, respectively.^1^ However, in Iranian men and women, 2.2 and 1.8 per 100 000 people per year were computed, respectively.^10^ In general, in the Iranian population, lip and oral cancer with 10 139 new cases and 454 deaths are ranked 20th and 22nd for morbidity, and mortality among all cancers.^9^

The availability of a system for tracking and giving precise information on the incidence of different cancers is one of the crucial elements of the cancer control program. The establishment of the Iran Cancer Registry system dates back to 1984, with the aim of creating a cancer registry at the national level, which would collect and analyze information from various hospitals and health facilities across the country.^11^ Cancer control policies and cancer epidemiological research in developed countries owe much to cancer registries and accurate cancer statistics of incidence and mortality. The lack of coverage and quality of cancer registration programs in the country resulted in various conflicting reports from pathology centers, and cancer registration centers regarding the incidence and prevalence of various cancers.

For more efficient planning and management of cancer prevention initiatives, it is vital to be informed of the existing status and trends in cancer incidence. There have been some comprehensive investigations of the incidence of different malignancies in Iran. As far as we know, there has not been a systematic review or meta‐analysis conducted in Iran to evaluate the incidence and trend of oral cavity malignancies. Therefore, the objective of this study is to conduct a thorough review and meta‐analysis of the age‐standardized incidence rate of oral cavity malignancies in Iran.

## 2 METHODS

As advised by the MOOSE (Meta‐analyses of Observational Studies in Epidemiology) Checklist,^12^, ^13^ this systematic review was carried out. This checklist contains instructions on how to present observational epidemiology research that has undergone meta‐analysis. The approach is covered in six parts, including the introduction, search strategy, methods, results, discussion, and conclusions. There are additional in‐depth assessment questions inside each component. Besides, the executive protocol of this study is registered in the international prospective register of systematic reviews PROSPERO 2022 CRD42022374132 Available from: https://www.crd.york.ac.uk/prospero/display_record.php?ID=CRD42022374132.

### 2.1 Search strategy

**FIGURE 1:**
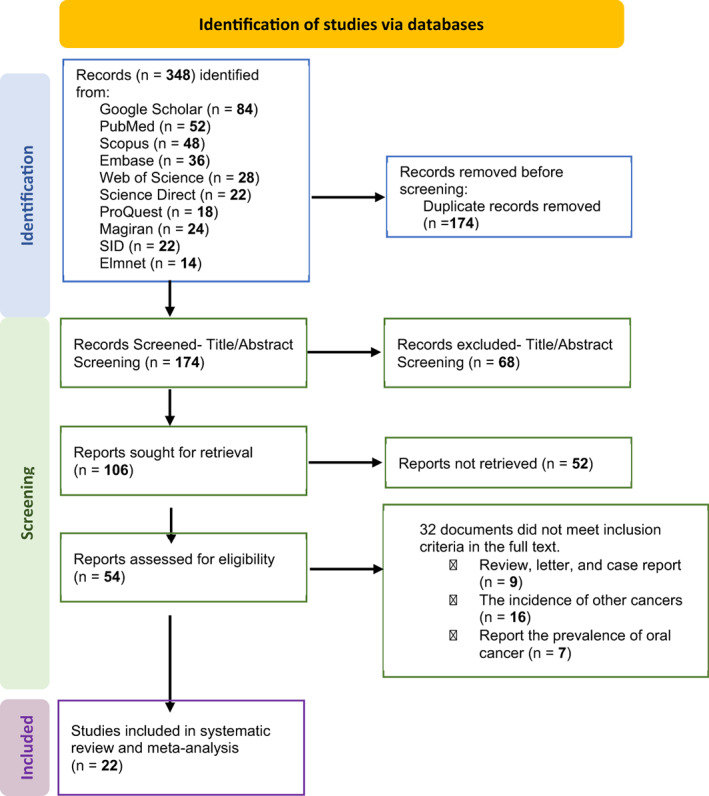
PRISMA flowchart of studies included in this systematic review and meta‐analysis.

The researchers searched for relevant articles without a time limit till December 2022 in Iranian databases SID (Scientific Information Database), Magiran and element as well as international databases PubMed/MEDLINE, Web of Science, ScienceDirect, Embase, Scopus, ProQuest, and Google Scholar. Additionally, the reference list of the discovered papers was manually reviewed to boost the search's sensitivity. Using ICD‐0‐3 codes, the team determined that the oral cavity consists of the lip (Vermillion, mucosal surfaces, both commissures), tongue, mouth floor, gum, hard palate, mouth, buccal mucosa, and vestibule. The search method was performed using MeSH terms in combination or separately using “AND,” and “OR” functions (Supplementary Table 1). The search process and the process of selecting related articles are shown in the PRISMA flowchart (Figure 1).

### 2.2 Inclusion and exclusion criteria

The review included studies that provided clear descriptions of the age‐standardized rate (ASR) of oral cavity cancer and contained reports from populations in Iran. Studies with limited sample numbers and those that reported prevalence were excluded. The final meta‐analysis did not take into account review studies or publications presented as conference posters.

### 2.3 Data extraction

The reviewers were aware of the name of the publication and the authors to gather information from relevant papers. The age‐standardized incidence rate (ASR), which was taken from the publications and put into this form, the sample size, sex, and the time and place of the research were all included in the data collecting form, which was an Excel sheet that was constructed based on the study's goals. The authors were contacted for access to additional data, and the evaluation of the articles for which the full text was not available.

All articles were assessed for duplicates. They were then chosen based on the titles and abstracts of linked papers. To evaluate the quality of the studies, two impartial reviewers assessed the entire text based on the inclusion and exclusion criteria. The cases of disagreement between two referees were independently evaluated, and resolved by the third referee (Table 1).

**TABLE 1:** The article met the eligibility criteria of this systematic review and meta‐analysis.

Order	Authors name	Years of publication	Period	Location	Sample size	ASR (males)	ASR (females)	QA
1	Mousavi et al.^36^	2009	2003–2004	Iran	641	1.18	1.18	5
			2004–2005	Iran	650	1.34	1.09	
			2005–2006	Iran	605	1.08	1.25	
2	Almasi et al.^37^	2016	2012	Iran	1380	2.2	1.8	7
3	Mohebbi et al.^38^	2017	2012	Iran	1380	2.2	1.8	7
4	Fallah et al.^39^	2007	2002	Iran	1063	2.9	1.7	6
5	Chamani et al.^23^	2009	1991–2002	Kerman	668	2.41	1.34	7
6	Kujan et al.^40^	2017	2012	Iran	1380	2.2	1.8	8
7	Khanali et al.^41^	2021	2000	Iran	1806	3.38	2.37	8
			2016	Iran	1925	2.66	2.16	
8	Rad et al.^25^	2009	1991–2002	Kerman	569	1.64	0.4	8
9	Mohagheghi et al.^32^	2009	1998–2001	Tehran	570	2.6	2.4	5
10	Mehrabani et al.^26^	2008	1990–2005	Fars	3115	0.58	0.29	6
11	Somi et al.^28^	2009	2006–2007	East Azerbaijan	2018	1.67	3.58	5
12	Sadjadi et al.^30^	2003	1996–1999	Ardabil	3455	3.8	1.7	5
13	Babaei et al.^33^	2005	1998–2002	Semnan	1732	1.95	3.43	5
14	Somi et al.^29^	2008	2006–2007	East Azerbaijan	4912	3.58	2.78	7
15	Mirzaei et al.^42^	2016	2003–2009	Iran	4993	9.7	9	6
16	Rabiei et al.^34^	2016	2004–2005	Guilan	1575	0.81	0.72	7
			2005–2006	Guilan	2045	1.62	1.29	
			2006–2007	Guilan	1426	1.41	0.43	
			2007–2008	Guilan	2142	1.33	1.89	
			2008–2009	Guilan	2436	1.91	1.25	
17	Talaiezadeh et al.^35^	2013	2002–2009	Khuzestan	16 801	0.02	0.02	8
18	Babaei et al.^31^	2009	2004–2006	Ardabil	4413	0.6	0.2	8
19	Roshandel et al.^43^	2014	2012	Iran	ND	2.2	1.8	5
20	Masoompour et al.^27^	2011	1998–2002	Fars	8359	0.1	0.1	7
21	Iranfar et al.^44^	2016	2008	Iran	1518	2.5	1.8	7
22	Sadjadi et al.^24^	2007	1996–2000	Kerman	5884	1.4	0.9	8

Abbreviations: ND, no data; QA, quality assessment.

### 2.4 Quality assessment (risk of bias)

The risk of bias (internal validity) of the included studies was evaluated using the modified Newcastle Ottawa scale for observational study checklist,^14^, ^15^ which assigns a score ranging from 0 to 10. Based on the scores obtained for each study using the checklist, the risk of bias was classified into three categories: low risk (scores of 6–10), medium risk (scores of 3–5), and high risk (scores of 0–3).^15^, ^16^

### 2.5 Statistical analysis

The meta‐analysis was performed using Stata version 16 software. The researchers assessed the heterogeneity using statistical techniques, such as the *I* ^2^ statistic and Cochran's *Q* test. When no heterogeneity was detected, a fixed‐effect model was used. However, in cases of heterogeneity, a random‐effect model was utilized.^17^, ^18^ The random‐effects model was used to extend the study's findings beyond the included studies by assuming that the selected studies are random samples from a larger population.^19^ The researchers employed multivariable meta‐regression analysis to estimate the effects of potential factors in heterogeneity.^18^

The sensitivity analysis approach, which involved one‐by‐one elimination, was used to investigate the influence of each study on the pooled incidence estimate. The researchers assessed the robustness of each model and ultimately selected the best mode.^14^, ^20^

The forest plot was used to display the overall age‐standardized rate (ASR) and its corresponding 95% confidence intervals (CI). To assess publication bias, Egger's and Begg's tests were performed. Additionally, the trim‐and‐fill approach was employed to determine the number of missing studies and adjust the total estimate.^21^, ^22^

## 3 RESULTS

### 3.1 Search results and eligibility studies

Three local databases (*n* = 24 from Magiran, *n* = 22 from SID, and *n* = 14 from Elmnet) and seven international databases (*n* = 84 from Google Scholar, *n* = 52 from PubMed, *n* = 48 from Scopus, *n* = 36 from Embase, *n* = 28 from Web of Science, *n* = 22 from Science Direct, and *n* = 18 from ProQuest) combined to identify 348 studies. The next step. The titles and abstracts of 120 articles and 174 duplicate articles that failed to fulfill the inclusion criteria were removed. Moreover, 32 papers were disqualified from meeting the inclusion criteria, encompassing reviews, letters, case reports, prevalence of other cancers, and accounts of oral cancer prevalence in the complete text. Figure 1 shows that a total of 22 publications were incorporated in the meta‐analysis and systematic review (Figure 1).

### 3.2 Characteristics of the eligible studies

The study comprised 22 journal papers in total. The NOS criteria were used to evaluate the risk of bias, and the results showed that six of the studies had a moderate risk of bias, while 16 of the studies had a low risk of bias (Table 1). Regarding the study location, three studies were conducted in Kerman,^23^, ^24^, ^25^ two studies in Fars,^26^, ^27^ two studies in East Azerbaijan,^28^, ^29^ two studies in Ardabil,^30^, ^31^ one study in Tehran,^32^ Semnan,^33^ Gilan,^34^ and Khuzestan,^35^ and nine studies were conducted in Iran^36^, ^37^, ^38^, ^39^, ^40^, ^41^, ^42^, ^43^, ^44^ (Table 1).

### 3.3 The results of individual studies

Based on the findings of included studies, the ASR ratio for oral cancer of men to women for oral cavity cancer was 1.20.

The highest ASR for males was recorded during 2003–2009 with a rate of 9.7 per 100 000. In Ardabil, the ASR was 3.8 per 100 000 between 1996 and 1999, while in East Azerbaijan, it was 3.58 per 100 000 between 2006 and 2007. On the other hand, the lowest ASR for males was reported in Khuzestan between 2002 and 2009 with a rate of 0.02 per 100 000 and in Fars between 1998 and 2002 with a rate of 0.1 per 100 000.

The highest ASR for females was reported during 2003–2009, with a rate of 9 per 100 000. East Azerbaijan reported a rate of 3.58 per 100 000 between 2006 and 2007, and Semnan reported a rate of 3.43 per 100 000 between 1998 and 2002. The lowest ASR for females was reported in Khuzestan between 2002 and 2009 (0.02 per 100 000), Fars between 1998 and 2002 (0.1 per 100 000), and Ardabil between 2004 and 2006 (0.2 per 100 000) (Table 1).

### 3.4 The pooled ASR of oral cavity cancer in male

**FIGURE 2:**
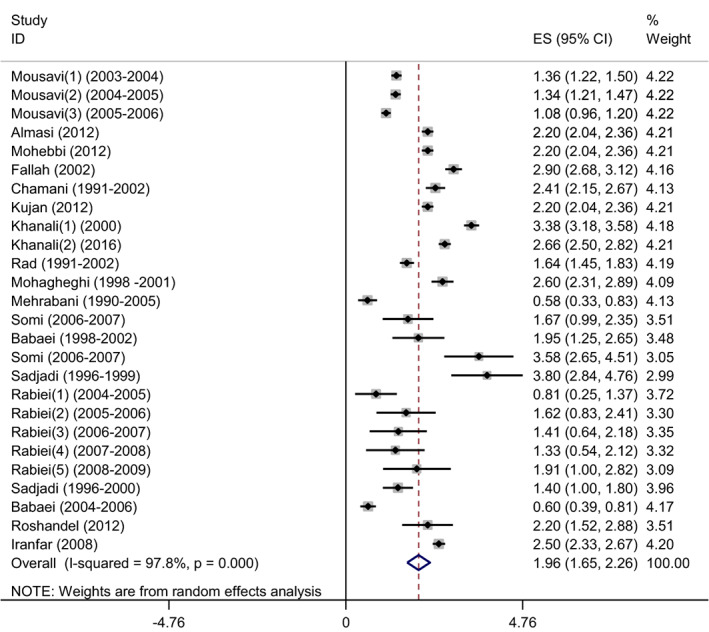
Forest plot age‐standardized incidence rate (ASR) of oral cavity cancer among males in Iran with sensitivity analysis using one‐by‐one elimination of studies method.

We estimated pooled ASR of oral cavity cancer 1.96 (95% CI: 1.65–2.26) (*Q* statistic = 1118.09, df = 25, *p* < .0001, *I* ^2^ = 97.8%), with random effect model after sensitivity analysis using one‐by‐one elimination of studies and the best robustness model was chosen among the males (Figure 2).

Because the *Q* statistic and *I* ^2^ = 97.8% were significant, indicating high heterogeneity among the studies, univariate and multivariable meta‐regression analyses were utilized to determine the source of the heterogeneity.

Furthermore, meta‐regression analysis demonstrated that geography, bias risk, research year, and sample size are not likely sources of heterogeneity (*p* > .05) (Table 2).

**TABLE 2:** Univariate and multivariable meta‐regression to find possible causes of heterogeneity between studies included in the meta‐analysis.

ASR	Possible cause of heterogeneity	Univariate		Multivariable	
		Coefficient (95% CI)	*p*‐value	Coefficient (95% CI)	*p*‐value
Male	Location	−0.19371 (−0.39682, 0.00939)	.062	−0.05086 (−0.16903, 0.06730)	.399
	Risk of bias	−0.27183 (−0.80202, 0.25835)	.315	−0.03217 (−0.29546, 0.23110)	.811
	Year	0.02361 (−0.02717, 0.07439)	.362	0.02253 (−0.02122, 0.06629)	.313
	Sample size	−0.00016 (−0.00034, 9.7806)	.064	−0.00014 (−0.00032, 0.00004)	.136
Female	Location	−0.14963 (−0.36337, 0.06409)	.135	−0.01973 (−0.11338, 0.07391)	.680
	Risk of bias	−0.42246 (−0.91700, 0.07207)	.094	−0.17801 (−0.38740, 0.03137)	.096
	Year	0.04281 (0.00154, 0.08407)	.042	0.04517 (0.01140, 0.07894)	.009
	Sample size	−0.00015 (−0.000311, −3.9306)	.044	−0.00013 (−0.00027, 0.00001)	.076

### 3.5 The pooled ASR of oral cavity cancer in female

**FIGURE 3:**
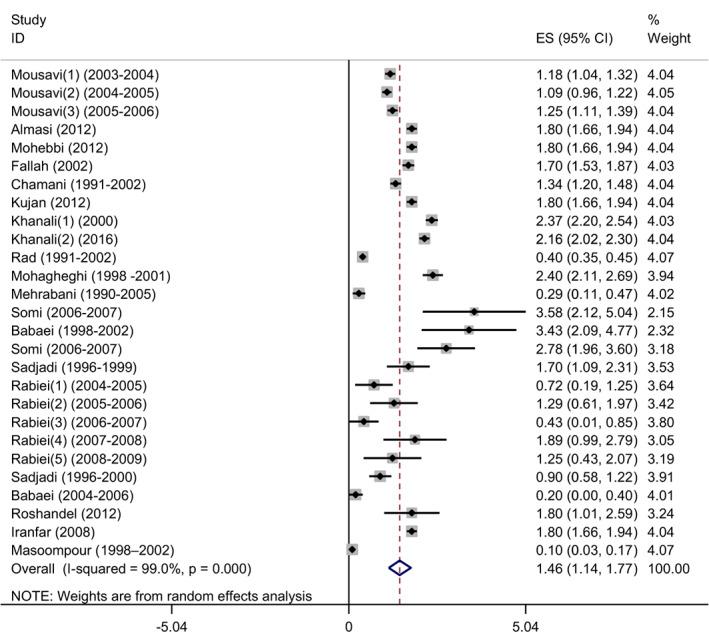
Forest plot age‐standardized incidence rate (ASR) per 100 000 people of oral cavity cancer among females in Iran with the sensitivity analysis using one‐by‐one elimination of studies method.

After performing sensitivity analysis using one‐by‐one elimination of studies, a random effect model was used to estimate the pooled age‐standardized incidence rate (ASR) of oral cavity cancer for females, which was determined to be 1.46 (95% CI: 1.14–1.77) (*Q* statistic = 2576.99, df = 26, *p* < .0001, *I* ^2^ = 99.0%) (Figure 3).

The high heterogeneity among the studies was observed based on the significant *Q* statistic and *I* ^2^ = 99%. To identify the source of heterogeneity, univariate and multivariable meta‐regression analyses were employed.

Through a univariate meta‐regression analysis, it was found that sample size studies (*p* = .044) and the study period (*p* = .042) were potentially responsible for the observed heterogeneity among the studies. A multivariable meta‐regression also suggested that the study period may have contributed to the data's heterogeneity (*B*‐coefficient = 0.04517, *p* = .009). As a result, altering the study period can increase the incidence by 0.04517. In a multivariable analysis, the location, sample size, or bias risk of the studies did not show a significant difference between them (with *p* value <.05).

### 3.6 Publication bias

To evaluate publication bias, Egger's regression test and funnel plots were utilized. In the case of verified publication bias, the trim‐and‐fill approach was applied to estimate the number of missing studies and adjust the pooled estimate from the meta‐analysis.^14^, ^22^

**FIGURE 4:**
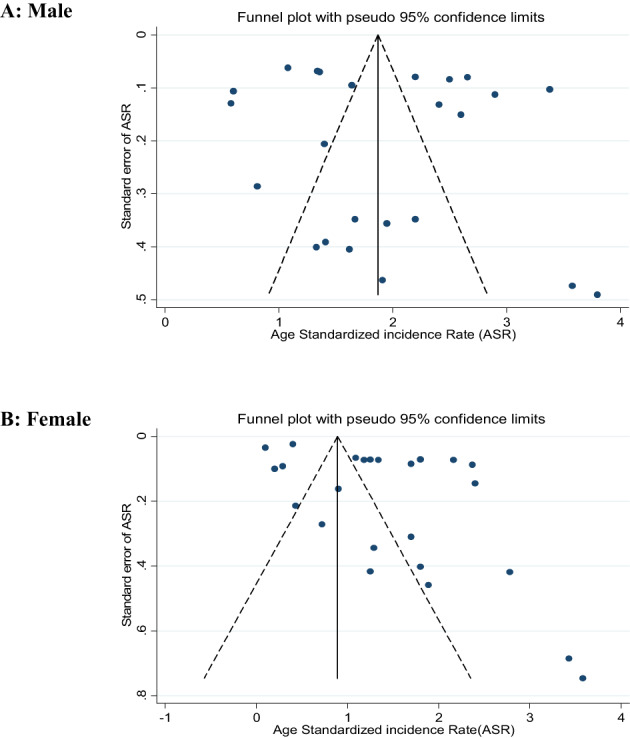
Funnel plot with pseudo 95% confidence limits for the detection of publication bias among included studies.

According to the results of the funnel plots and Egger's test, there is no evidence of publication bias in studies reporting on oral cavity cancer in males (bias = 1.3220, 95% CI: −3.9571, 6.6012, *p* = .610) (Figure 4A).

For ASR of the oral cavity cancer in females, the funnel plot was asymmetric and Egger's test was significant (bias = −7.6366, 95% CI: 2.2141, 13.05904, *p* = .008) (Figure 4B).

Based on Trim‐and‐fill analysis using non‐parametric methods, the anticipated values of three censored studies were calculated, and overall ASR corrected by the random effects model was calculated to be 1.36 (95% CI: 1.05%–1.66%) in females.

## 4 DISCUSSION

In Iran, cancer ranks third as a cause of mortality.^45^ According to Globocan 2020, lip and oral cancer are ranked 20th and 22nd for morbidity, and mortality among cancers in the Iranian population.^9^ The variation in the incidence rate of cancer between Globocan's report and Iran's report may be due to differences in estimates and sources of information in Iran. Globocan data, which provides estimates of cancer incidence, mortality, and prevalence at the global, regional, and national levels, is calculated using a combination of data sources and mathematical modeling. To date, there has been no systematic review or meta‐analysis carried out in Iran to evaluate the incidence of oral cavity cancers and compare their incidence and trends.

The findings of this study revealed that the age‐standardized rate (ASR) for oral cavity cancer is 1.96 and 1.36 per 100 000 for Iranian men and women, respectively. According to GLOBOCAN 2020, lip and oral cavity cancer had incidence rates of 6 and 2.3 per 100 000, respectively, on a global scale. Melanesia had the highest ASR (22.2 for men and 11.9 for females), followed by south‐central Asia (13.3 for males and 4.6 for females). Central America, western Africa, and northern Africa have the lowest ASR: 1–1.7 for men and 1.1–1.3 for females. Iran is situated in western Asia, and the ASR for the nations in this area indicated male mortality rates of 2.2 and female mortality rates of 1.3. The findings of this research were near the ASR range for western Asian nations, notably for the female group, which was 1.36. Oral cancer stands among 6th most prevalent malignancies in the world.^46^ Even though oral cavity and lip cancer were not listed in the top 10th, in Iran^47^ some studies reported its increasing trend.^42^, ^48^, ^49^ On one hand, Oral cancer occurs more in the elderly.^50^ On the other hand, the population profile in Iran will have an increasing trend in individuals more than 65 years old.^40^ This means that as time goes on, oral cavity cancer may become an even more concerning issue than ever. It is estimated that the projection of cancer incidence by 2030 would be doubled in comparison to 2012.^51^

Similar to the majority of other reports, the ASR ratio for oral cancer showed dominance in men. The reason for the higher incidence of oral cancer in men compared to women could be due to their increased exposure to risk factors, including smoking, alcohol consumption, tobacco use, and outdoor activities that increase exposure to solar radiation. Our result showed a 1.2 age‐standardized incidence rate (ASR) ratio for oral cancer of men to women, however, various numbers are reported in other studies for instance in Taiwan men were predominantly 15 times more than women.^52^

In some particular high‐risk countries in South Asia like Indonesia^53^ and Singapore,^54^ women were at the same risk as men. Some justifications for this diversity might be in terms of differences in risk factors exposure, behavioral, cultural, dietary habits, occupational, and environmental factors.

In both genders, East‐Azerbaijan province stands among the top 2 with the highest ASR rate. Moreover, Ardebil as the neighboring province to East‐Azerbaijan, both located in the northwest of Iran, has the highest ASR rate for males. Considering both provinces count as high‐risk regions for gastrointestinal cancers, particularly esophageal and gastric cancers,^55^ possible overlap in risk factors for G.I. cancers and oral cancers may be the reason for high ASR rates^56^, ^57^ Furthermore, the population of both provinces is from the same ethnicity, race, which have the same dietary habit^29^ may have eventuated in the high rate of ASR.

According to the reports, the age‐standardized incidence rate (ASR) of all cancers has been found to be higher in the northern countries of the West Asia region, including the north of Iran, when compared to the southern countries of the region, which includes the south of Iran.^43^ The same trend was observed, as the least amount of ASR rate is reported in Fars and Khuzestan provinces. Furthermore, accessibility to medical facilities varies in different provinces which leads to this verity in ASR incidence rate.

Unlike most of the world^58^ Alcohol consumption cannot be taken as the main risk factor in Iran,^23^ because it is forbidden due to local regulations. However, tobacco specifically abuse in the form of a water pipe can be considered one of the most notable risk factors.^59^

Our study had several limitations. First, Although the study shows that the ASR incidence rate for oral cavity cancer in Iran is lower than in the world, taking into the fact that it is reported that all cancers in Iran are underestimated,^39^ the ASR incidence rate for all cancers, including oral cavity cancer, might be more than what is reported. Age‐standardized rates have certain limitations that are widely recognized, such as being used as relative indices for comparison purposes only and not providing precise measurements of actual rates when the age compositions of groups are different. Third, in this study, the heterogeneity index was very high. Despite performing subgroup analysis and meta‐regression to deal with heterogeneity, Some of the remaining heterogeneity can be due to variables that were not investigated in our studies such as improvements in medical care and care systems over time and new diagnostics being made available.

Increasing awareness to avoid the risk factors exposure, and the development of screening programs will reduce the burden of this disease and its incidence rate.^60^ A well‐structured cancer registry system and subsequent studies are suggested to follow the future ASR incidence rate and ascertain more risk factors in Iran.

## 5 CONCLUSION

In Iran, the incidence of oral cavity cancer was lower than the world average. However, we expect an upward trend, as a result of an upward trend in life expectancy, the number of elderly, and various exposures to risk factors, such as smoking. To detect cancer at an early stage, a cancer registry system, and screening programs should be developed.

## AUTHOR CONTRIBUTIONS

**Mohammad Jokar:** Data curation (equal); investigation (equal); methodology (equal); software (equal); visualization (equal); writing – original draft (equal); writing – review and editing (equal). **Negin Namavari:** Data curation (equal); methodology (equal); validation (equal); writing – original draft (equal). **Seyed Alireza Moshiri:** Data curation (equal); investigation (equal); writing – original draft (equal). **Hossein Kargar Jahromi:** Data curation (equal); investigation (equal); visualization (equal). **vahid rahmanian:** Conceptualization (equal); formal analysis (equal); investigation (equal); methodology (equal); project administration (equal); software (equal); supervision (equal); validation (equal); writing – original draft (equal); writing – review and editing (equal).

## CONFLICT OF INTEREST STATEMENT

The authors have stated explicitly that there are no conflicts of interest in connection with this article.

## ETHICS STATEMENT

In this study, we adhered to all ethical principles in the systematic review and meta‐analysis studies. The Research Ethics Committee of Jahrom University of Medical Sciences approved the study protocol (ID IR.JUMS.REC.1401.112.).

## TRANSPARENCY STATEMENT

The lead author (Vahid Rahmanian) affirms that the manuscript presents a comprehensive, truthful, and clear account of the study. The study plan was adhered to, and any deviations were elucidated, and if necessary, documented.

## Supporting information


**Supplementary table 1** Strategy search for the incidence of oral cavity cancer in Iran.

## Data Availability

The data that support the findings of this study are available from the corresponding author upon reasonable request.
