# Supplementary material for: The incidence of oral cavity cancer in Iran: A systematic review and meta‐analysis
Source: Cancer Rep (Hoboken). 2023 May 16;6(6):e1836. doi: 10.1002/cnr2.1836 (PMC10242657; doi:10.1002/cnr2.1836)
Supplement: Supplementary file 1 — Supplementary table 1 Strategy search for the incidence of oral cavity cancer in Iran. [file CNR2-6-e1836-s001.docx]

S1 Table: strategy search for the Incidence of Oral Cavity Cancer in Iran

| **Databases** | **Search terms** |
| --- | --- |
| Google Scholar, Scopus, PubMed/MEDLINE, Science Direct, Web of Science, EMBASE, ScienceDirect, ProQuest  SID (Scientific Information Database), Magiran, element, | (Mouth Neoplasm) OR(Neoplasm, Mouth) OR(Neoplasms, Oral)(Oral Neoplasm) OR(Cancer of Mouth) OR (Mouth Cancer) OR (Oral Cancer) OR(Cancer of the Mouth), OR (Cancer, Mouth) OR (oral squamous cell carcinoma), (cavity oral cancer )OR (oral carcinoma)OR(oral malignant)OR(oral tumor)OR(oral growth) OR (Lip Neoplasm) OR (Cancer of Lip) (Lip Cancers) OR (Cancer, Lip) OR (Cancers, Lip) OR (Lip cancer) OR(Tongue Cancer) OR (Tongue Neoplasm) OR ( Cancer of Tongue) OR ( gum cancer) OR ( Vestibule cancer)OR(cancer of the buccal mucosa) and “incidence” or epidemiology or age-standardized rate or ASR and “Iran. |
| **Number of paper identified** | **Results in each database** |
| 348 journal papers | Google Scholar=84 papers  PubMed=52 papers  Scopus=48 papers  Embase=36 papers  Web of Science=28 papers  Science Direct=22 papers  ProQuest=18 papers  Magiran= 24 papers  SID=22 papers  Element=14 papers |
